# The availability and delivery of culturally responsive Australian Aboriginal infant resuscitation education programmes: a structured literature review

**DOI:** 10.1017/S1463423623000373

**Published:** 2023-08-07

**Authors:** Nakita Stephens, Caroline Nilson, Tracy Reibel, Rhonda Marriott

**Affiliations:** 1 Murdoch University, College of Health and Education - School of Nursing, Mandurah, Western Australia, 6210, Australia; 2 Ngangk Yira Institute for Change, Murdoch University, Murdoch, Western Australia, 6150, Australia

## Abstract

**Aim:**

To critically appraise the literature to determine availability and identify the cultural responsiveness of infant resuscitation education for Aboriginal and Torres Strait Islander populations.

**Background:**

Despite overall reductions in infant mortality in the last two decades, Aboriginal people have some of the highest rates of infant mortality of any developed nation. One of the key factors that has attributed to improvements in infant mortality rates is parent and carer education around risk factors and actions of first responders. Identifying gaps in the current basic first-aid initiatives available to Aboriginal communities may contribute to developing resources to contribute to reductions in Aboriginal neonatal mortality rates.

**Method:**

The review used key terms and Boolean operators across an 11-month time frame searching for research articles utilising the databases of CINAHL, Scopus, Ovid Emcare, Informit, Pubmed and Proquest. After review, 39 articles met the inclusion criteria, 25 articles were discarded due to irrelevant material and 14 articles were included in the structured literature review. The search process was developed using the Preferred Reporting Items for Systematic Reviews and Meta-Analysis guidelines. Articles were assessed for validity and inclusion using the Critical Appraisal Skills Program checklist.

**Results:**

Research literature relating to First Nation community-based CPR and first-aid education programmes in Canada, USA, India, UK and Europe, Asia and Africa were identified; however, none pertaining specifically to CPR and first-aid education in Australian Aboriginal communities were found.

**Discussion:**

Despite the lack of research evidence relating to infant cardiopulmonary resuscitation (CPR) education for Australian Aboriginal populations, the reviewed studies noted the importance of culturally responsive education designed in collaboration with First Nation peoples, using novel ways of teaching CPR, that align with the language, culture and needs of the communities it is intended for.

**Conclusion:**

Further research is required to create a framework for the delivery of culturally responsive infant resuscitation education for Australian Aboriginal parents and communities.

What does this paper add to the wider global clinical community?

- The importance of providing culturally responsive education to Aboriginal communities, which are co-designed and community-specific.
- Review of novel ways for infant resuscitation education for parents.

Note: for conciseness the term Aboriginal will be used to refer to both Aboriginal and Torres Strait Islander peoples with no disrespect intended.

## Background and significance

Aboriginal and Torres Strait Islander people have some of the highest rates of infant death in Australia, including sudden unexpected death in infancy (SUDI) and sudden infant death syndrome (SIDS). In 2018, data from the Australian Mothers and Babies Report (Australian Institute of Health & Welfare, 2020) stated that 1 in 18 or 5.7% of all babies born in Australia were Aboriginal, and of those more than one-quarter (28%) were admitted to a special care nursery (SCN) or a neonatal intensive care unit (NICU), and one-fifth of those required active resuscitation. Babies born to Aboriginal mothers are 1.6 times more likely to be admitted to SCN/NICU, 14% are born prematurely and 12% are low birth weight. The reasons for these statistics are complex and include socio-economic and broader social, cultural, environmental and political factors, and the ongoing effects of colonisation (Australian Institute of Health & Welfare, 2020) which likely contribute to poorer outcomes. Aboriginal neonatal death rates in 2018 were 4.6 deaths per 100 births, representing a small decrease from 6.2 deaths per 1000 in 2008 (Australian Institute of Health & Welfare, 2020). Of all Aboriginal infant deaths, 13% were classified as ‘signs, symptoms and abnormal clinical child deaths not classified elsewhere including SUDI and SIDS’ (Commonwealth of Australia, 2020). In the period 2014–2018, 514 of the 603 (85%) Aboriginal deaths were infants under one year of age (Commonwealth of Australia, 2020). The Closing the Gap Report (Commonwealth of Australia, 2020) reported that during 2018, Aboriginal infants were 1.8 times more likely to die than non-Indigenous infants (5.1 compared with 2.9 per 1000 live births). While the proportion of Aboriginal infant deaths occurring out-of-hospital is not reported, there has been an identified need to address the issues of community competence and awareness in cardiopulmonary resuscitation (CPR) and first aid in Western Australia (Celenza *et al.*, 2002, Lynch *et al.*, 2005).

The odds of survival from cardiac and/or respiratory arrest are improved by two to three times when bystanders commence CPR (Goto *et al.*, 2014, Bergamo *et al.*, 2016, Weinmeister *et al.*, 2018). Dispatchers in emergency centres are increasingly instructing callers on how to perform CPR; however, it takes an average of 1 minute longer to commence CPR when being instructed compared with people who already know CPR (Goto *et al.*, 2014). It has also been noted that the emotional state of the caller can also adversely affect their ability to follow the CPR instructions given by dispatchers (Weinmeister *et al.*, 2018). One of the steps in the chain of survival is early CPR which can greatly improve the outcomes of out-of-hospital arrests (Goto *et al.*, 2014, Weinmeister *et al.*, 2018). In a study exploring incidents of CPR given by bystanders, it was noted that children received bystander CPR in only around a third to one half of out-of-hospital arrests (Goto *et al.*, 2014). A large cohort study in the USA examined 5807 incidences of CPR in the under 18 age groups, with 68% occurring in the under 1 age group (Knudson *et al.*, 2012). The study identified a higher survival rate in this age group (44%), further demonstrating the increased likelihood of survival with prompt infant CPR (Knudson *et al.*, 2012). The World Health Organisation’s stance on prompt CPR is based on several studies that demonstrate favourable outcomes (Bergamo *et al.*, 2016). Providing bystander CPR in out-of-hospital-cardiac arrest (OHCA) greatly increases survival; however, bystander CPR is only provided in less than half (46%) of OHCAs (Bergamo *et al.*, 2016). A Swedish study exploring bystander witnessed cardiac arrest also noted improved survival in the under 12-month age group with prompt CPR (Gelberg *et al.*, 2015). Many infants who have a respiratory arrest will respond to prompt CPR, and given that parents/carers are usually the first to find an unresponsive child, their skills in CPR are critical (Arnold and Diaz, 2016).

Infant CPR is not often taught as part of the mainstream parenting education in the perinatal period despite a number of studies exploring this need, and presently there are no standards for discharge education in Australian NICUs (Arnold and Diaz, 2016). When education is offered, it often differs between institutions, and resources such as mannequins and trained staff are often limited (Arnold and Diaz, 2016, Murray and Joseph, 2016). One study in an American NICU that required CPR training of all parents prior to discharge noted that of the 102 discharges during the study period only 37 parents of infants had learned infant CPR (Murray and Joseph, 2016); therefore, 63% of families did not have or receive CPR training. The timing of infant CPR education is also important because stress and anxiety levels as well as fear and sadness during or soon after admission can affect the ability to learn new information (Arnold and Diaz, 2016). Other factors such as feed times and work schedules need to be taken into consideration as they too can impact on the delivery of effective education and training (Murray and Joseph, 2016). However, there are increasing requests from parents of infants who are ‘graduating’ home from the NICU to upskill other family carers in infant CPR because of the higher risk for aspiration, choking and cessation of breathing in this cohort of infants (Murray and Joseph, 2016). It has also been noted that parents of infants who did not require extra assistance post-birth also wanted education on how to resuscitate their babies should something occur (Stephens, 2019). Given the rural and remote location of many Australian Aboriginal communities, timely emergency service access may be variable dependant on the season or distance; therefore, first responders may be parents or members of the community. Whilst there are some organisations that offer online infant CPR resources for Australian Aboriginal parents to review (Royal Life Saving NSW, 2013, Raising Children Network, 2020), computers, internet connectivity, IT proficiency and literacy may be some of the barriers to accessing these resources. Celenza and colleagues (2002) note that some CPR is better than no CPR and attempts by non-professional community members may improve survival. The authors also recommend that training authorities develop CPR training to meet the needs of the specific target groups that emphasise those skills that are easily remembered and can be practised frequently and are more likely to be implemented in the real-life situation.

A study conducted by Naim *et al.* (2019) in the USA noted clear racial and ethnic disparity in whether CPR was provided by a bystander with a disproportionate amount of cardiac or respiratory arrests happening in low-income neighbourhoods. Similar findings were noted within lower socio-economic groups in Taiwan (Tzeng *et al.*, 2021). This also impacted on the time it took for dispatchers to recognise that CPR was required, and this was thought to be related to language barriers and lack of language proficiency amongst this lower socio-economic group as well as lower CPR awareness in this community (Tzeng *et al.*, 2021). Lower rates of CPR training is more common in some countries, as a study in Greece identified that only 6.5% of the general population in a county region had received training in the last 12 months (Konstandinos *et al.*, 2008). In a study conducted in sub-Saharan Africa, the majority of the communities not only had limited awareness of CPR but also lacked the capacity and infrastructure to encourage, deliver or facilitate CPR education (Kalu *et al.*, 2018). Kalu and colleagues (2018) noted that both healthcare professionals and the general public needed training in CPR, noting the importance of the bystanders involvement leading to improved outcomes (Kalu *et al.*, 2018). A study conducted in Ghana noted that 90% of 479 survey respondents had never undertaken CPR training, and 25.4% of the 190 ambulance workers surveyed had also not completed any training in relation to resuscitation (Anto-Ocrah *et al.*, 2020). The study concluded that 90% of all respondents would be interested in CPR training (Anto-Ocrah *et al.*, 2020). A study conducted in Zambia noted the importance of tailoring first-aid training to those who are receiving it after using emergency first-aid training developed in South Africa. The researchers noted that due to the differences of these two countries, many of the educational subjects were not relevant to the situations faced in Zambia (Pigoga *et al.*, 2017). This demonstrates the importance of tailoring first-aid and resuscitation education for the people receiving it. This was noted in a case study conducted in Queensland, Australia, with an Aboriginal community in Toomelah. Here, tailored education was provided to equip the community to respond to medical emergencies (Anderson, 2006). The case study demonstrated the importance of responding to cultural protocols, citing the agreement that was made between the community and the regional emergency service to turn off the ambulance lights and sirens at the bridge leading into the community due to embarrassment felt by community members as it was described as causing a ‘big spectacle’ when a triple zero emergency call was made. This increased the number of triple zero calls made by the community when an emergency arose, and the collaboratively designed training ensured that community members were upskilled in first aid to act as first responders. The case study highlighted a scenario in which the life of a young boy was saved as a result, and it further demonstrates that programmes developed collaboratively with communities can make a difference (Anderson, 2006).

### Search strategy

Informed and guided by the Preferred Reporting Items for Systematic Reviews and Meta-Analysis (PRISMA) guidelines, the review was conducted between the end of November 2020 and the end of September 2021 utilising the databases CINAHL, Scopus, Ovid Emcare, Informit, Pubmed and Proquest. The search strategy involved the use of the following Boolean operators ‘Resus* OR CPR * OR Cardiopulmonary Resuscitation* OR Infant resus* OR first aid OR basic life support’ AND ‘Infant* OR baby OR Neonate* OR Newborn’ AND ‘education OR training OR cultural programs’ AND ‘Aboriginal parents OR Aboriginal mothers OR Indigenous Parents OR Indigenous Mothers OR Aboriginal carers or Indigenous carers OR Aboriginal communities OR Indigenous communities’. Boolean operators were used to make the search more precise whilst still remaining broad enough to capture the required data.

### Article selection

A total of 550 articles were initially found using the search strategy criteria, and 18 others were found from additional sources during sequel searches. After an initial review and the removal of duplicates, a total of 160 articles remained. These first pool articles were reviewed, and a further 121 articles were further excluded for not meeting criteria (see Table 1). To fit the purpose of this structured review, studies were included if they (1) discussed the study’s design, (2) were published in English and from the year 2000 onwards, (3) discussed infant CPR or basic life support (BLS), (4) discussed community-based CPR or BLS or first-aid programmes and (5) were available in full text. Only peer-reviewed published studies and official reports were included in this review. A full manuscript review was undertaken of the remaining 39 articles, and one article was eliminated because it was not reporting a research study, and eight articles did not report infant CPR data; 10 articles reported on an irrelevant age group; four articles contained data that was prior to the year 2000; and two articles were not able to be secured in full-text form. The 14 articles that remained met the inclusion criteria, used a range of study designs, and were published between 2010 and 2020 (see Table 2).

**Table 1.**
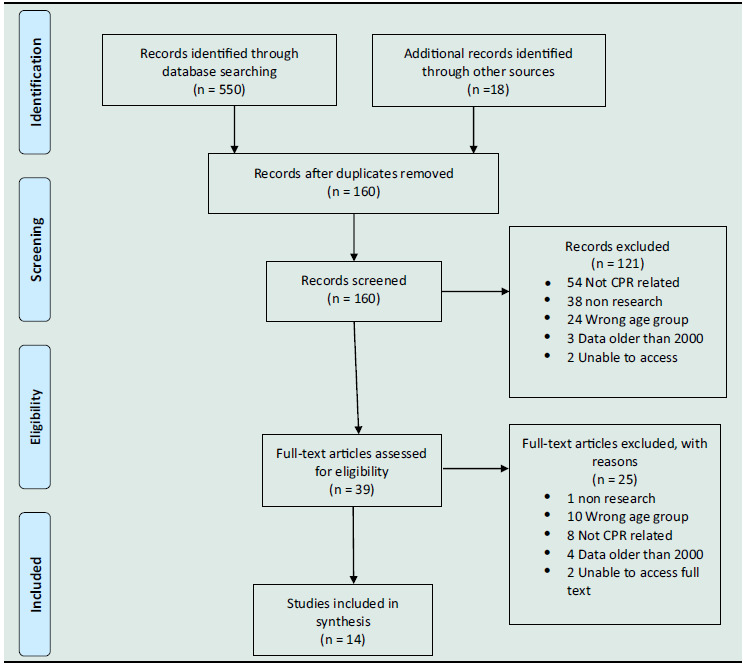
PRISMA flow diagram: infant CPR systematic review

**Table 2.** CASP checklists

Author	Clear statement of aims	Appropriate methodology used	Was the design fitting to address the aims	Was the recruitment strategy appropriate	Was the data collected in a way that addressed the aims	Was the data analysis rigorous enough	Is there a clear statement of findings	Can the results be applied to the local people	Do the results fit with other available evidence	Totals (yesnNo/ can’t tell)
Barker *et al.*	Y	Y	Y	Y	Y	Y	Y	Y	Y	9/0/0
Barry	Y	Y	Y	Y	Y	Y	Y	Y	Y	9/0/0
Barry *et al.*	Y	Y	Y	Y	Y	Y	Y	Y	Y	9/0/0
Bergamo *et al.*	Y	Y	Y	Y	Y	Y	Y	Y	Y	9/0/0
Chia *et al.*	Y	Y	Y	Y	Y	Y	Y	Y	Y	9/0/0
Curran *et al.*	Y	Y	Y	Y	Y	Y	Y	Y	Y	9/0/0
Guo *et al.*	Y	Y	Y	Y	Y	Y	Y	Y	Y	9/0/0
Hollenberg *et al.*	Y	Y	Y	Y	Y	Y	Y	Y	Y	9/0/0
Katona *et al.*	Y	Y	Y	Y	Y	Y	Y	Y	Y	9/0/0
Knight *et al.*	Y	Y	Y	Y	Y	Y	Y	Y	Y	9/0/0
Mew *et al.*	Y	Y	Y	Y	Y	Y	Y	Y	Y	9/0/0
Orkin *et al.*	Y	Y	Y	Y	Y	Y	Y	Y	Y	9/0/0
Pierick *et al.*	Y	Y	Y	Y	Y	Y	Y	Y	Y	9/0/0
Yip *et al.*	Y	Y	Y	Y	Y	Y	Y	Y	Y	9/0/0

### Data extraction and quality assessment

The 14 articles that were included for the final review were appraised and assessed for validity using the Critical Appraisal Skills Program (CASP) checklists. Specific CASP study design checklists were used for the differing study designs, and the results against the appraisal criterion are presented in Table 2. The CASP checklists identified the suitability of the aims, methodology, design, participant/subject recruitment and data collection, and the rigour in the data analysis and presentation of the findings, with a scoring schema of ‘yes’, ‘no’ or ‘cannot tell’. Two reviewers appraised the studies (NS and CN), and any discrepancies in the quality assessment were resolved through consensus. Once the appraisal was complete, the individual checklist tables were synthesised and drawn together into a presentation comparison table (see Table 3). Extracted variables for the presentation table include author(s), year of the publication, study design, location of study setting, aims/objectives, participants, outcomes and limitations. Details of the protocol for this structured review were registered on PROSPERO and can be accessed at   https://www.crd.york.ac.uk/PROSPERO/display_record.php?RecordID%20=%20222 316

**Table 3.** Article comparison table

Author	Year	Design	Location	Aims	Participants	Outcomes	Limitations
Barker & Ross	2014	Retrospective case review	Northern Territory, Australia	To describe the remote paediatric aeromedical population in the Northern Territory	789 participants under the age of 16 years	Age, gestation if newborn, diagnosis at referral, requirement for high-dependency care and transport team members	Results could have been more expansive when presented but is only a short article.
Barry	2015	Mixed methods survey	Limerick, Ireland	Evaluate knowledge, satisfaction and use of a 22-minute self-instructional infant CPR kit	77 participants, including 42 nulliparous women and their partners	Pre-test and post-test knowledge scores and satisfaction with kit	The immediate pre- and post-tests were conducted in a controlled setting; however, the 6-month follow-up test was not and therefore parents could have reviewed the information prior to the test.
Barry, Dixon, Armstrong & Keane	2019	Prospective cohort study	Ireland	To determine the effectiveness and acceptability of providing self-instructional training via an e-learning programme coupled with the use of a novel infant pillow mannequin	44 participants	Confidence in knowing what to do with a choking infant, confidence in performing infant CPR, ability to perform skill, perceived acceptability of the infant pillow mannequin to learn infant CPR	Small sample size, log-in details used to determine the level of use; however, parents could have shared log-ins. Questionnaires and responses were not matched with physical assessment. Assessment conducted on the pillow mannequin, so transferability of skills has been questioned.
Bergamo, Bui, Gonzales, Hinchey, Sasson & Cabanas	2016	Retrospective observational analysis	America	Would targeted CPR training in neighbourhoods with low bystander CPR and high incidence of cardiac arrests increase the incidence of bystander CPR for adult OHCA	11 242 community members	Increased incidence of CPR in OHCA and whether more people in high-risk zip codes would be educated	Only conducted in one US city did not analyse whether compression only CPR improved survival outcomes
Chia & Lian	2014	Mixed methods questionnaire	Singapore	To survey the knowledge, attitudes and perceptions of parents regarding infant basic life support	375 parents of NICU infants	Basic infant knowledge level, attitudes towards learning and performing infant CPR	Did not appear to be any content validity undertaken on the questionnaire prior to administration to participants.
Curran, Ritchie, Beardy, VanderBurgh, Born, Lewko & Orkin	2018	Qualitative community-based participatory research	Sachigo Lake, Canada	To understand the characteristics of medical emergencies and how medical emergencies are managed in remote Indigenous community	12 members of Sachigo Lake First Nation, 12 other members of local community (24 participants in total)	Description of local health emergencies and the local response to emergencies	Small sample size and only qualitative data collected. Quantitative data could have been collected as well.
Guo & DeCoux Hampton	2021	Pre-test/post-test questionnaire	America	To increase CPR knowledge through providing training in a native language	47 participants with low English proficiency	CPR knowledge in the community increased, and there was considerable interest in continuing the training	Small sample size, only two classes run with the first being very small and the second growing in size
Hollenberg, Claesson, Ringh, Nordberg, Hasselqvist-Ax, & Nord	2018	Sub-group analysis of a randomised trial	Sweden	To investigate whether students’ native language affects CPR skills or willingness to act	641 13-year-old students	Total score directly after training and 6 months after training	Lack of understanding of other native language students’ skills in Swedish and the use of different instructors
Katona, Douglas, Lena, Ratner, Crothers, Zondervan & Radis	2015	Pre-test/post-test questionnaire	South Sudan	To use the wilderness first-aid course to increase first-aid knowledge	46 participants	Improvement in knowledge in the post-test questionnaire	Small sample size, conducting the course in English with live translation may have affected some of the understanding
Knight, Wintch, Nichols, Arnolde & Schroeder	2013	Observational survey	America	Examine the efficacy of the CPR Anytime kit as a standardised method for learning infant CPR	117 parents of high-risk infants	Surveys at 1, 3 and 6 months, satisfaction with the kit, knowledge level, comfort with infant CPR, kit dissemination	Smaller sample size that continually decreased with each follow-up. Those that remained until the 6 months follow-up were noted to have completed formal CPR training previously.
Mew, Ritchie, VanderBurgh, Beardy, Gordon, Fortune, Mamakwa & Orkin	2017	Environmental Scan involving community-based participatory research	Nishnawbe Aski Nation (NAN), Ontario, Canada	To understand emergency response systems, services and training in remote NAN communities	10 First Nation community health leaders and 33 other key stakeholders (43 participants in total)	Response services and capacity, volunteer emergency services and challenges to emergency care	Small sample size and only qualitative data collected
Orkin, VanderBurgh, Born, Webster, Strickland & Beardy	2012	Community-based participatory research	Sachigo Lake First Nation, Canada	Through collaboration can culturally responsive first-aid education be created for the community	20 members of the Sachigo Lake First Nation community	Community-based first response training programme in remote Indigenous communities	Limited to only one community’s involvement
Pierick, Waning, Patel & Atkins	2012	Prospective observational cohort study	America	Asses the usefulness of self-instructional DVD kit for families of high-risk infants	311 parents of high-risk infants	Comfort level with performing CPR, kit dissemination and use during the study	Limited questionnaire of only around five questions that determined comfort with the kit. ‘Comfort’ was not defined.
Yip, Ong, Tu, Chavez, Ike, Painter, Lam, Bradley, Coronado & Meischke	2010	Qualitative methodology using focus groups	America	To determine the current level of knowledge in relation to CPR in the lower English proficiency community	46 participants	To determine current knowledge levels and if participants knew where to access education	Small sample size did not seem to test their CPR knowledge but rather what they knew about CPR

## Results

A total of 14 studies were included in the review. Several study types were noted which included one retrospective case study (7%), one retrospective observational analysis (10%), five surveys/questionnaires (35%), two prospective cohort studies (15%) and three community-based participatory research methods (22%), one randomised trial (7%) and one focus group (7%). The studies were conducted in Australia (7%), Ireland (14%), Singapore (7%), America (36%), Canada (22%), Sweden (7%) and South Sudan (7%). Outcomes ranged from the diagnosis and need for high-dependency transport (7%) to knowledge and attitudes to learning infant CPR (36%), teaching high-risk populations first aid (14%), rural medical issues (21%) and native language training (21%). In those studies that utilised an infant CPR education kit, outcomes ranged from knowledge levels pre- and post-kit use and satisfaction with the kit (50%) to confidence in skills and ability to perform resuscitation skills (17%). Of the four studies that reviewed infant CPR education kits, 75% used the CPR Anytime™ training Kit and 25% used the pillow CPR kit and e-learning programme. Further information, including aims and limitations of the studies, are included in Table 3. It appears that there is a paucity of literature regarding Australian Aboriginal perceptions of infant CPR, because no articles specific to this topic were found.

A retrospective study conducted in Australia reviewing the need for aeromedical high-dependency transport described the cohort of 789 cases as being predominantly Aboriginal (Barker and Ross, 2014). The most common causes of transport were respiratory, with bronchiolitis and pneumonia at 31%, and the mean age range for transportation with bronchiolitis was 0.7 years (0.1–6.8) (Barker and Ross, 2014). The researchers noted that the majority of transport cases were newborns, which included preterm infants occurring in higher rates amongst the Aboriginal population. The increased risks and needs for high-dependency intervention amongst Aboriginal infants suggest a need for education for Aboriginal caregivers surrounding infant CPR. Another study was conducted in the USA (Bergamo *et al.*, 2016) and used a retrospective observational analysis to determine whether the implementation of a short compression only CPR course provided in high-risk post codes would lead to increased CPR given by bystanders. The uptake was positive with a total of 11 242 participants trained in the TAKE10 compression only method. The provision of the course focused on cultural safety by engaging community members to deliver the education. The course implementation was deemed a feasible way to increase the provision of CPR by bystanders (Bergamo *et al.*, 2016), which could potentially be used as a blueprint for culturally responsive education for Aboriginal parents.

Several studies were conducted in remote locations of Canada involving first-aid education with Indigenous communities. Curran and colleagues (2018) explored the management of medical emeregencies in remote First Nation communities. The findings identified that remote communities often lacked an established coordinated response system, and that there was a need to create education that was culturally specific and sustainable for the individual community and the region it was to be used in (Curran *et al.*, 2018). Twenty-four community members participated in the Sachigo Lake Wilderness Emergency Response Education Inititive traing course (SLWEREI). Despite a low recruitment rate that impacted the generalisability of the results, the participant cohort had a high interest rate in learning remote area first aid. The major barriers to appropriate management of medical emergencies identified in this study included the lack of access to culturally appropriate health services, or formal community response systems, the complexity of emergencies experienced in remote areas, and the inexperience of local first responders (Curran *et al.*, 2018). The study by Orkin *et al.* (2012) also looked at the specific challenges faced by First Nation peoples living a traditional life in the remote wilderness. Using a community-based participatory research approach, 20 Sachigo Lake First Nation people were involved in the SLWEREI training course mentioned previously. The irrelevance of generic first-aid courses in remote settings which focus on patient stabalisation while awaiting almost immediate assistance and transporation were highlighted. The findings identified the need for relevant remote medical emergency training to increase the community’s confidence to provide care that could lead to improved outcomes for remote populations. Similar issues were identified in a study conducted with 10 Nishnawbe Aski Nation health leaders and 33 other stakeholders, to review their emergency response systems (Pierick *et al.*, 2012). Issues ranged from having inconsistent emergency service dispatch systems to the complete absence of a system and inconsistent service coverage not spanning 24 h a day. The lack of trained community first-aid responders was also highlighted, identifying the need for community-based first-aid training.

In South Sudan, a wilderness first-aid course was designed to teach people with minimal first-aid experience in order to increase the chances of bystander intervention in an emergency, especially given the lack of a formal emergency medical service in the area (Katona *et al.*, 2015). This course was specifically designed for the South Sudanese area, and community collaboration informed the correct language that should be used in the teaching tools. The programme’s culturally sensitive user manuals, posters and questionnaires were developed with consideration of the general literacy level of the community and were all picture-based (Katona *et al.*, 2015). Through this study, it was found that first aid could be effectively taught to people with varying levels of education and experience and was found to be promising for rural communities in particular (Katona *et al.*, 2015).

A number of studies focused on the language used to train participants. A study conducted in Sweden where CPR training is mandatory in schools looked at whether using a CPR instruction video in Swedish or in participants’ native language such as Bosnian/Croatian/Serbian/English and Persian would make a difference to their skills ability and knowledge retention (Hollenberg *et al.*, 2019). A total of 641 students were analysed, and the study found that students whose first language was not Swedish scored considerably less on the practical tests when learning from the Swedish language video (Hollenberg *et al.*, 2019). It was also noted that limited skills in the Swedish language was also a barrier to calling emergency services in a timely manner for an emergency situation (Hollenberg *et al.*, 2019).

A further two studies documented CPR knowledge and training in Chinese communities with limited English proficiency (LEP) in the USA. One study conducted by Yip *et al.* (2011) in Seattle, Washington, noted that CPR training was predominantly offered in English and was less available to communities with LEP. As the training is mostly conducted in English, there was limited awareness of CPR knowledge in the Chinese community, and very few community members had actually received instruction (Yip *et al.*, 2011). The study’s findings identified the use of local media to advertise programmes, to increase community involvement and to equip community members with skills to increase CPR bystander uptake. Further, the findings supported the delivery of CPR programmes in varying dialects of Chinese (Cantonese and Mandarin) (Yip *et al.*, 2011). The second study conducted by Guo and Hampton (2021) in the San Francisco Bay Area noted that although there are many various CPR training programmes offered to the community, relatively few are offered to communities with LEP. It has been noted that language barriers lead to significant delays in the recognition and commencement of CPR, even with dispatcher assistance, and also leads to poorer performance of CPR due to these difficulties (Guo and Hampton, 2021). By offering CPR courses in the participants’ native language, the Chinese LEP community felt that they would be able to use these resources to learn and understand CPR processes, and there was great interest from the community to continue the programmes (Guo and Hampton, 2021).

To determine parent’s knowledge, attitudes and perceptions of infant basic life support, Chia and Lian (2014) recruited 400 parents of infant patients in the neonatal department of a Singapore hospital. Of the total parents enrolled, 375, only 55% had passed the basic knowledge section of the questionnaire and only 26% had received previous CPR training (Chia and Lian, 2014). This could indicate an issue with the questionnaire itself as no content validity assessment could be determined from the article, whereas other studies utilised previously tested specific CPR knowledge questions. It was also noted that those who had received previous training received lower total knowledge scores with only 35% passing (Chia and Lian, 2014). The vast majority of parents both previously trained and untrained wanted to attend an infant CPR course or refresher (Chia and Lian, 2014). Amongst those who were not interested in basic life support education, the most common reason was the lack of time to attend CPR classes (Chia and Lian, 2014). This is an important point to note, because the remaining articles in this systematic review included self-taught infant CPR kits that do not require class attendance and can be done at the participant’s convenience.

Barry *et al.* (2019) utilised an e-learning programme in conjunction with a pillow mannequin. The pillow mannequin has the outline of an infant’s face and body, with the nose and mouth circled on the front of the pillow slip and the back of an infant on the back of the pillow slip. The slip would fit any standard size pillow and was a lower cost option than other CPR kits. The e-learning programme involves two videos, one on infant basic life support and one on infant choking. Participants are encouraged to watch these videos numerous times and then practice their skills on the pillow mannequin. This study looked at participants’ confidence, skills and comfort of use with the programme. There was a considerable increase in participants’ confidence in performing CPR and what to do when an infant was choking from 25% prior to the programme to 88% of participants after the programme (Barry *et al.*, 2019). Of those whose skills were assessed, the vast majority indicated the correct position for a choking infant. However, CPR skills such as checking for the chest rising were far lower in those assessed (Barry *et al.*, 2019). Overall participants felt that this type of programme was simple, useful and easily accessible for busy parents (Barry *et al.*, 2019).

Many of the articles focused on knowledge levels, confidence, satisfaction and use of at home infant CPR kits. The CPR Anytime™ training kit is not currently available in Australia; however, it can be ordered online. It was produced by the American Heart Association and includes a DVD, instructions, and an inflatable mannequin to practise the skills on. As this kit is self-instructional and does not require class attendance, it can be undertaken anytime that is suitable to new parents as well as being used to educate other members of the family who may look after the infant.

Pierick *et al.* (2012) enrolled 311 participants including 238 parents of premature infants and 73 parents of infants with congenital heart disease and aimed to assess the usefulness and parent comfort associated with the infant CPR Anytime™ kit. This is a particularly high-risk group of infants more likely to need infant CPR. It was noted that 75% of participants had received prior CPR education before receiving the kit (Pierick *et al.*, 2012). It was determined that parents’ comfort levels with performing infant resuscitation increased with continued use of the kit and participants shared the kit with other family members particularly in the first four months of this study (Pierick *et al.*, 2012). This study did not seek to determine if knowledge level or skills improved with use of the kit. However, the study did note that eight incidents occurred during the study period requiring intervention by parents with skills they had learned from the kit. Importantly participants noted that using the kit was reassuring to them as they were aware of their infant’s higher risk status (Pierick *et al.*, 2012). One participant felt that the kit was useful and should be given to all parents, including parents of healthy infants not just at-risk infants (Pierick *et al.*, 2012).

Knight, Wintch, Nichols, Arnolde and Schroeder provided the infant CPR Anytime™ kit to 117 parents of high-risk infants prior to discharge from the hospital, who were then contacted at one, three and six months to assess retention of knowledge and skills and comfort with using the kit (Knight *et al.*, 2013). This study noted that all participants adequately demonstrated CPR skills at the initial assessment (Knight *et al.*, 2013). A few participants were not contactable to enable follow-up over the course of the study period; however, those with prior CPR training were more likely to respond to subsequent follow-ups which could affect the results. In the follow-up reviews of 86, 73 and 61 participants at one, three and six months, respectively, in relation to the knowledge levels and skills in performing CPR, there was an identified increase on CPR skills performance and an increase in knowledge of what to do in an emergency situation (Knight *et al.*, 2013). There were minimal changes between the one-, three- and six-month scores noted in both knowledge and confidence development. Participants also noted that they had shared the kit with at least one family member if not more, leading to family members also being educated in infant CPR (Knight *et al.*, 2013).

Barry (2015) enrolled 77 participants which included pregnant women and their partners, who were provided with the CPR Anytime™ kit and tested pre-training, post-training and at six-month post-training. The study also sought to determine if the participants would share the kit with other family members who may look after the infant. All participants felt that other family members should use the kit; however, only 45 other family members accessed the kit during the study period (Barry, 2015). It was noted that the knowledge scores increased as predicted in the post-training test and remained higher than pre-training test at six months (Barry, 2015). This indicates good retention of the skills and knowledge learned from the CPR Anytime™ kit. This study also found that participants’ confidence in providing infant CPR improved as did the number of people who said they would provide CPR in an emergency situation (Barry, 2015).

## Discussion

The main purpose of this structured review was to critically appraise the literature to determine availability and identify the cultural responsiveness of infant resuscitation education for Australian Aboriginal populations. Despite an extensive literature search, only one non-research focused case study was found addressing a community-based first-aid programme in an Australian Aboriginal community (Anderson, 2006). While it was excluded for not meeting the review criteria, it highlights a gap in the literature. The most likely cause for emergency aeromedical transport in Aboriginal communities is for respiratory illness in infants (Barker and Ross, 2014). This is important as it is also noted that many infant respiratory arrests can be completely reversed (Parsons and Mackinnon, 2009, Weinmeister *et al.*, 2018). Some Aboriginal parents are known to delay seeking treatment for their infants due to the belief that their infant is likely be sent far away to receive treatment. Further, that treatment may not be culturally responsive or allow for the inclusion of traditional bush medicine (Kruske *et al.*, 2012). Highlighting this gap in the literature is also important given the Aboriginal infant mortality rate that was reported between 2014 and 2018 to be high, noting that 85% of the Aboriginal deaths that occurred where in the under one year age group (Commonwealth of Australia, 2020). Studies conducted in Canada with Aboriginal communities demonstrate that community involvement in providing culturally responsive first-aid education is well received by the community and encourages community resourcefulness (Orkin *et al.*, 2012, Vanderburgh *et al.*, 2014, Mew *et al.*, 2017, Curran *et al.*, 2018) and demonstrates an increased need for community-specific infant resuscitation education particularly in remote areas (Orkin *et al.*, 2012, Mew *et al.*, 2017, Curran *et al.*, 2018). Further, CPR education should also be tailored to suit the needs of the community receiving it and should be presented in numerous languages for comprehension and retention of information (Yip *et al.*, 2011, Katona *et al.*, 2015, Hollenberg *et al.*, 2019,, Guo and Hampton, 2021). Additionally, when education is presented in the participants’ native language dissemination in the community improves, leading to increased knowledge of CPR and an increase in community members wanting to be trained (Katona *et al.*, 2015, Guo and Hampton, 2021). These articles further support the need to gain a better understanding of what Australian Aboriginal parents want and need in their infant CPR training.

Many of the reported OHCAs that occurred in homes involved infants, and bystander CPR has been shown to increase the odds of survival two to three times, supporting the idea that teaching parents CPR could save lives (Knight *et al.*, 2013, Bergamo *et al.*, 2016). However, no standard or protocol for infant CPR discharge education exists in Australia at present and is therefore not conducted as routine in many NICUs/SCNs (Arnold and Diaz, 2016). A number of studies have noted that teaching parents infant CPR leads to increased confidence, feelings of self-efficacy and decreased anxiety as well as saving lives (Pierick *et al.*, 2012, Knight *et al.*, 2013, Barry, 2015, Barry *et al.*, 2019). The timing of discharge education is also noted to be very important because fear, anxiety, stress and sadness affect a person’s ability to learn new information (Arnold and Diaz, 2016). Therefore, there is an advantage in the use of self-instructional, at home CPR education allowing parents to undertake the training at a time and place that is most suitable to them. This can be done several ways including e-learning programmes or the use of the infant CPR Anytime™ kit. The most favourable appears to be the infant CPR Anytime™ kit which was used in a number of studies. Comfort with the kit was an important aspect of many studies, and it was determined that parents found the kit acceptable, easy to use and beneficial, and the kit was often shared with other family members (Pierick *et al.*, 2012, Knight *et al.*, 2013, Barry, 2015). However, the kit is not currently available in Australia, although it can be ordered online. Adapting the kit for use with the Aboriginal population might be an answer to provide education to remote communities in particular. Another option presented was the use of a pillow manikin. Although this is an excellent low-cost idea for parents to be educated in infant CPR, using different pillow sizes may lead to some confusion in relation to the depth of the required chest compressions. It should be noted that any education concerning infant CPR for Aboriginal communities must be developed in consultation and collaboration with those communities to ensure that the programme is culturally safe and responsive to their cultural needs (Price-Robertson and McDonald, 2011, Orkin *et al.*, 2012, Vanderburgh *et al.*, 2014).

## Conclusion

Providing infant CPR education to parents will likely lead to the development of appropriate skills, improved confidence and decreased levels of anxiety to act as first responders. Bystander CPR is a critical step to ensure survival with good neurological outcomes, and there can be a delay in commencing CPR if bystanders need to be instructed by dispatchers. The use of self-instructional infant CPR kits provide education at a time that is beneficial to parents. The paucity in the literature regarding Aboriginal parents’ perceptions of infant CPR and the lack of availability of culturally specific infant CPR training programmes in Australia supports the need for collaborative and consultative research into these areas. Utilising these methods and engaging and collaborating with Australian Aboriginal communities to develop a framework for infant resuscitation education that is culturally responsive to Aboriginal parents’ needs and adaptable to a variety of settings could save lives and provide parents, carers and communities with important skills. Evaluation should also be conducted on the provision of such culturally sensitive infant CPR education to determine its effectiveness and if such education impacts on infant mortality and morbidity rates, particularly in remote communities.
